# High-molecular-weight hyaluronan – a possible new treatment for sepsis-induced lung injury: a preclinical study in mechanically ventilated rats

**DOI:** 10.1186/cc6982

**Published:** 2008-08-08

**Authors:** Yung-Yang Liu, Cheng-Hung Lee, Rejmon Dedaj, Hang Zhao, Hicham Mrabat, Aviva Sheidlin, Olga Syrkina, Pei-Ming Huang, Hari G Garg, Charles A Hales, Deborah A Quinn

**Affiliations:** 1 Pulmonary and Critical Care Unit, Department of Medicine, Massachusetts General Hospital, 55 Fruit Street Boston, MA 02114, USA; 2 Harvard Medical School, 25 Shattuck St, Boston, MA, 02115 USA; 3 Chest Department, Taipei Veterans General Hospital, Sec 2, Shih-Pai Rd, Taipei, 11217, Taipei, Taiwan; 4 National Yang-Ming University, School of Medicine, No.155, Sec.2, Linong Street, Taipei, 112 Taiwan; 5 Department of Internal Medicine, National Cheng Kung University Hospital, 138 Sheng-Li Road, Tainan, 70428 Taiwan; 6 Genzyme Corporation, 500 Kendall Street, Cambridge, MA 02142 USA; 7 Shriners Burn Hospital, 51 Blossom Street, Boston, MA 02114 USA; 8 Department of Traumatology and Surgery, National Taiwan University Hospital, No. 7, Chung-Shan S. Road, Taipei 100, Taiwan

## Abstract

**Introduction:**

Mechanical ventilation with even moderate-sized tidal volumes synergistically increases lung injury in sepsis and has been associated with proinflammatory low-molecular-weight hyaluronan production. High-molecular-weight hyaluronan (HMW HA), in contrast, has been found to be anti-inflammatory. We hypothesized that HMW HA would inhibit lung injury associated with sepsis and mechanical ventilation.

**Methods:**

Sprague–Dawley rats were randomly divided into four groups: nonventilated control rats; mechanical ventilation plus lipopolysaccharide (LPS) infusion as a model of sepsis; mechanical ventilation plus LPS with HMW HA (1,600 kDa) pretreatment; and mechanical ventilation plus LPS with low-molecular-weight hyaluronan (35 kDa) pretreatment. Rats were mechanically ventilated with low (7 ml/kg) tidal volumes. LPS (1 or 3 mg/kg) or normal saline was infused 1 hour prior to mechanical ventilation. Animals received HMW HA or low-molecular-weight hyaluronan via the intraperitoneal route 18 hours prior to the study or received HMW HA (0.025%, 0.05% or 0.1%) intravenously 1 hour after injection of LPS. After 4 hours of ventilation, animals were sacrificed and the lung neutrophil and monocyte infiltration, the cytokine production, and the lung pathology score were measured.

**Results:**

LPS induced lung neutrophil infiltration, macrophage inflammatory protein-2 and TNFα mRNA and protein, which were decreased in the presence of both 1,600 kDa and 35 kDa hyaluronan pretreatment. Only 1,600 kDa hyaluronan completely blocked both monocyte and neutrophil infiltration and decreased the lung injury. When infused intravenously 1 hour after LPS, 1,600 kDa hyaluronan inhibited lung neutrophil infiltration, macrophage inflammatory protein-2 mRNA expression and lung injury in a dose-dependent manner. The beneficial effects of hyaluronan were partially dependent on the positive charge of the compound.

**Conclusions:**

HMW HA may prove to be an effective treatment strategy for sepsis-induced lung injury with mechanical ventilation.

## Introduction

Hyaluronan (HA), an important component of the extracellular matrix, is composed of repeating disaccharide units containing alternating D-glucuronic acid and *N*-acetyl glucosamine. HA has been shown to produce distinct biological effects depending on the molecular weight. HA is synthesized by hyaluronan synthase (HAS) that is located in the cell membrane, and is secreted into the interstitial space [1]. In mammalian cell culture, HAS 1 and HAS 2 produce high-molecular-weight hyaluronan (HMW HA), whereas HAS 3 produces low-molecular-weight hyaluronan (LMW HA) [2,3].

HA has been identified as an important modulator in many physiological and pathological processes. Under physiological conditions, HA exists predominantly in the HMW HA form (>500 kDa), and maintains the structural integrity of the extracellular matrix in the lungs. In disease conditions during inflammation, LMW HA (<500 kDa) is produced either by depolymerization of HMW HA via oxygen radicals and enzymatic degradation by hyaluronidase, β-glucuronidase, and hexosaminidase or by *de novo *synthesis through HAS 3 [4].

LMW HA can function as an intracellular signaling molecule in inflammation and has been found to be proinflammatory [5,6]. We have found that LMW HA from stretched lung enhances IL-8 expression, and that LMW HA production by HAS 3 mediated ventilator-induced lung injury [7,8]. On the contrary, HMW HA can block inflammation. Transgenic HAS 2 mice that overexpress HMW HA have been found to be protected from bleomycin-induced lung injury [9]. We hypothesized that systemic administration of HMW HA would decrease sepsis-induced lung injury with mechanical ventilation by inhibiting cytokine production and lung inflammation.

## Materials and methods

### Animals

The present study was approved by the Massachusetts General Hospital Subcommittee on Research Animal Care. Sprague–Dawley viral-free rats, all in the growing phase, weighing between 185 and 225 g, were obtained from Charles River Laboratories (Wilmington, MA, USA).

### Ventilator protocol

The animals were anesthetized by intraperitoneal ketamine (90 mg/kg) (Abbott Laboratories, Chicago, IL, USA) and xylazine (10 mg/kg; Burns Veterinary Supply Inc., Rockville Centre, NY, USA) while breathing room air. Throughout the experiment, the animals were placed in a supine position on a heating blanket and the body temperature was monitored with a rectal probe. PE 240 tubing (outer diameter, 2.42 mm; internal diameter, 1.67 mm; Becton Dickson Infusion Therapy System Inc., Sandy, UT, USA) was inserted into the trachea and connected to a Harvard apparatus ventilator (model 55-7058; Harvard Apparatus, Holliston, MA, USA). The rats were then ventilated with a tidal volume of 7 ml/kg with a rate of 85 to 100 breaths per minute.

The end-tidal carbon dioxide pressure was monitored intermittently by a microcapnograph (Columbus Instruments, Columbus, OH, USA), and was maintained between 35 and 45 mmHg by adjusting the ventilator respiratory rate. The volume was increased by 5 ml/min to correct the air loss from the sample flow adaptor during monitoring of the end-tidal carbon dioxide. The peak inspiratory airway pressure was measured every 30 minutes with a pressure transducer amplifier (Gould Instrument System, Valley View, OH, USA) connected to the tubing at the proximal end of the tracheostomy.

The mean arterial pressure was measured every 30 minutes during mechanical ventilation using the same pressure transducer amplifier connected to PE 10 tubing (outer diameter, 0.61 mm; inner diameter, 0.28 mm; Becton Dickson Infusion Therapy System Inc., Sandy, Utah, USA) ending in the common carotid artery. During the period of ventilator use, intraperitoneal ketamine 0.05 mg/g and xylazine 0.005 mg/g were administered every 30 minutes, and 0.9% NaCl was infused, as needed, to maintain systolic blood pressure >90 mmHg. Harvesting of lung and bronchoalveolar lavage (BAL) was performed after 4 hours of mechanical ventilation.

### Model of lipopolysaccharide-induced lung injury with mechanical ventilation and pretreatment with hyaluronan

Sprague–Dawley rats were randomly divided into four groups: nonventilated control rats; mechanically ventilated rats with lipopolysaccharide (LPS) infusion as a model of sepsis; mechanical ventilation plus LPS infusion with HMW HA (1,600 kDa) pretreatment; and mechanical ventilation plus LPS infusion with LMW HA (35 kDa) pretreatment. Rats were mechanically ventilated with a low tidal volume (7 ml/kg) (n = 5 or 6 rats/group) for 4 hours. Rats received 3 ml of 0.35% of 1,600 kDa or 35 kDa (Genzyme Corp., Cambridge, MA, USA) pretreatment via the intraperitoneal route 18 hours prior to the beginning of study.

All HA preparations were sterile and were protein free and LPS free, to avoid known confounding effects of HA [10]. The size and amount of HA was chosen based on previous work that found HMW HA (>780 kDa) given intraperitoneally 18 hours before injection of concavalin protected against concavalin-induced liver toxicity. A dose response was found in this model, and 0.35% HMW HA was most effective [11]. We have shown that 1,600 kDa HA is the predominant size in normal rat lung [7]. Based on these findings, we used 0.35% of 1,600 kDa given intraperitoneally 18 hours prior to LPS infusion.

Rats received either 1 mg/kg *Salmonella typhosa *LPS (Lot 81H4018; Sigma Chemical Co., St Louis, MO, USA) or an equivalent volume of normal saline as control via the carotid artery. We have previously found arterial injection of LPS with mechanical ventilation to cause acute lung injury within 4 hours of injection. After 1 hour of spontaneous respiration to allow for development of a septic response, ventilation was begun. We used an established rodent model of mechanical ventilation as previously described [12-14]. Rats were sacrificed with an overdose of pentobarbital after 4 hours of ventilation. The left lung was lavaged with normal saline for measurement of cell counts and cytokines, macrophage inflammatory protein-2 (MIP-2) and TNFα. The right lung was flash frozen for the myeloperoxidase assay, for extraction of RNA for the measurement of HA synthase, and for determining the gene expression of cytokines, MIP-2 and TNFα. Separate groups of animals were used for determination of lung pathology.

### Model of lipopolysaccharide-induced lung injury with mechanical ventilation and therapeutic treatment with hyaluronan

Rats were ventilated in the same manner as described for the pretreatment with HA model. The 0.35% concentration of 1,600 kDa HA was too viscous for intravenous injection. For the studies post acute lung injury treatment, we performed dose–response studies with 0.025%, 0.05% and 0.1% of 1,600 kDa HA starting at the time of initiation of ventilation. Intravenous infusion of 0.1% sodium carboxymethyl cellulose (CMC) – a positive charged carbohydrate prepared from carboxymethylation of cellulose with a molecular weight of 1 × 10^5 ^to 7.5 × 10^5 ^– was used as a control condition for charge at 500 μl/hour. The infusion was continued throughout the experiment.

### Bronchoalveolar lavage

The lungs were removed *en bloc *and tubing was inserted into the trachea and secured. The right lung was clamped at the bronchus to prevent the lavage fluid from entering the right lung. The left lung was lavaged with 2 ml of 0.9% normal saline three times. One millilitre of the pooled effluents was used for cytospin and subsequent cell differentials, and 100 μl was used for the total cell counts. The remaining effluents were centrifuged at 3,000 rpm for 10 minutes, after which the supernatants were frozen at -80°C for further measurement of cytokines.

### Bronchoalveolar lavage cell counts

Neutrophil counts in BAL fluid were used to measure migration of neutrophils into alveoli and airways [15]. Total cell counts in BAL were performed using a hemocytometer. To measure cell differentials, the cells in the lavage fluid were fixed on glass slides with cytospin and were then stained with a hematologic stain kit (Fisher Diagnostics, Middletown, VA, USA).

### Myeloperoxidase assay

Myeloperoxidase activity in lung parenchyma was used as a marker of total lung neutrophil sequestration, including neutrophils marginalized in the vasculature, and in the interstitium and alveoli [13,15,16]. Samples of the right lower lobe were obtained within a few minutes of death and were stored at -80°C. The right lower lobe was thawed on ice, weighed, and homogenized in 5 ml phosphate buffer (20 mM, pH 7.4). One milliliter of the homogenate was centrifuged at 10,000 × *g *for 10 minutes at 4°C. The resulting pellet was resuspended in 1.0 ml phosphate buffer (50 mM, pH 6.0) containing 0.5% hexadecyltrimethylammonium bromide (Sigma Chemical Co.). The suspension was subjected to three cycles of freezing (on dry ice) and thawing (at room temperature), after which it was sonicated for 40 seconds and centrifuged again at 10,000 × *g *for 5 minutes at 4°C.

The supernatant was assayed for myeloperoxidase activity by measurement of hydrogen peroxide-dependent oxidation of 3,3',5,5'-tetramethylbenzidine (Sigma Chemical Co.). In its oxidized form, 3,3',5,5'-tetramethylbenzidine was measured by spectrophotometer at 650 nm. The reaction mixture for analysis consisted of 25 μl tissue samples, 25 μl 3,3',5,5'-tetramethylbenzidine (final concentration, 0.16 mM) dissolved in dimethylsulfoxide, and 200 μl hydrogen peroxide (final concentration, 0.30 mM) dissolved in phosphate buffer (0.08 M, pH 5.4) prior to adding to the mixture. The reaction mixture was incubated for 3 minutes at 37°C and the reaction stopped by adding 1 ml sodium acetate (0.2 M, pH 3.0), after which absorbance at 650 nm was measured. The absorbance followed a linear relationship with the myeloperoxidase concentration, which in turn is an enzyme marker for leukosequestration. The absorbance (*A*_650_) was reported as units (optical density) per gram of wet lung weight.

### Measurement of MIP-2 and TNFα in lavage fluid

Rat MIP-2 and TNFα were measured in BAL fluid using a commercially available ELISA kit containing antibodies that were cross-reactive with rats and mouse MIP-2 (BioSource International, Inc., Camarillo, CA, USA). Each sample was run in duplicate according to the protocol provided by the manufacturer.

### Isolation of RNA and measurement of mRNA expression by RT-PCR

For isolation of total RNA, the lungs were homogenized in 1.5 ml Trizol reagent (Invitrogen Life Technologies, Carlsbad, CA, USA) and were isolated according to the manufacturer's protocol. Total RNA (1 μg) was reversely transcribed into cDNA using a Gene Amp PCR system 9600 (PerkinElmer Life Sciences, Boston, MA, USA), as previously described [17].

The following primers of MIP-2 were used: PCR forward primer, 5'-TCC TCA ATG CTG TAC TGG TCC-3' and reverse primer, 5'-ATG TTC TTC CTT TCC AGG TC-3'; TNFα forward primer, 5'-CAT GAT CCG AGA TGT GGA ACT-3' and reverse primer, 5'-TCA CAG AGC AAT GAC TCC AAA G-3'; and GAPDH (internal control) forward primer, 5'-AAT GCA TCC TGC ACC ACC AA-3' and reverse primer, 5'-GTA GCC ATA TTC ATT GTC ATA-3' (Sigma Chemical Co.).

The following cycling parameters were used: MIP-2, denaturation at 94°C for 5 minutes followed by 35 cycles of 94°C for 30 seconds, annealing at 50°C for 45 seconds, and extension at 72°C for 30 seconds, with a terminal extension at 72°C for 7 minutes; and for TNFα, denaturation at 94°C for 5 minutes followed by 40 cycles of 94°C for 30 seconds, annealing at 58°C for 45 seconds, and extension at 72°C for 1 minute, with a terminal extension at 72°C for 7 minutes.

Results were quantified using densitometry. The GAPDH and cytokine signal densitometry was measured for each group. The cytokine signal was normalized to GAPDH expression and expressed as a ratio to control. A minimum of three mRNA samples were analyzed for each group.

### Pathology

After 4 hours of mechanical ventilation, the rats were sacrificed and the lung and trachea were removed. The left lung was infused at a pressure of 30 cmH_2_O with 10% buffered formalin, embedded in paraffin, sectioned at 4 μm thickness, and stained with hematoxylin and eosin. Ten randomly chosen fields in the parenchyma (without large airways) from the individual three lungs from each group were examined. Each of the pathological changes was scored on a scale of 0 to 3: 0 = alveolar filling, collapse or atelectasis (10×); 1 = inflammatory cell infiltration in the air space or vessel wall (20×); 2 = perivascular clubbing or swelling (10×); and 3 = alveolar hemorrhage or congestion (10×). The 10 randomly chosen fields at low power (10×) covered over 80% of the left lung. Because the injury was patchy, this technique gave an overview of the whole left lung. A higher power (20×) was needed to accurately identify inflammatory cell infiltration. The overall score was the sum of the average score for each category. Two subspecialists who were blinded to the treatment groups reviewed the degree of injury of each slides.

### Statistical methods

Analysis was performed using Statview 4.5 (SAS Institute Inc., Cary, NC, USA). All data are expressed as the mean ± standard error of mean. Analysis of variance for comparison of the different groups was used with significance set at *P *< 0.05. A significant analysis of variance was followed by a Fisher test for multiple comparisons between groups, significance set at *P *< 0.05.

## Results

### Systolic pressure, heart rate and airway pressure in ventilated rats with hyaluronan pretreatment

For animals in the experiments involving HA pretreatment, the systolic pressure was maintained with infusion of saline as needed to maintain systolic blood pressure >70 mmHg to minimize the confounding effects of hypotension. The amount of saline infused did not differ among groups. The peak airway pressures for all animals were between 8 and 12 mmHg. The systolic blood pressure did not differ among groups. The heart rate was increased in animals exposed to LPS but was not significantly different between treatment groups (Table 1).

**Table 1:** Hemodynamics in the pretreatment model

	Systolic blood pressure		Heart rate	
	
Group	Baseline	4 hours	Baseline	4 hours
Control	98 ± 7	85 ± 7	360 ± 7	327 ± 10
Lipopolysaccharide alone	100 ± 7	79 ± 5	436 ± 11	386 ± 11*
Lipopolysaccharide + 1,600 kDa hyaluronan	112 ± 7	96 ± 9	405 ± 64	412 ± 67*
Lipopolysaccharide + 35 kDa hyaluronan	100 ± 8	79 ± 7	369 ± 66	382 ± 65*

There were no statistical differences between groups for systolic blood pressure. In the lipopolysaccharide-exposed groups the heart rate was statistically higher, but there were no statistical differences between treatment groups. *P < 0.05 versus control.

### Pretreatment with HMW HA (1,600 kDa) completely blocked both lung neutrophil and monocyte infiltration induced by mechanical ventilation

**Figure 1:**
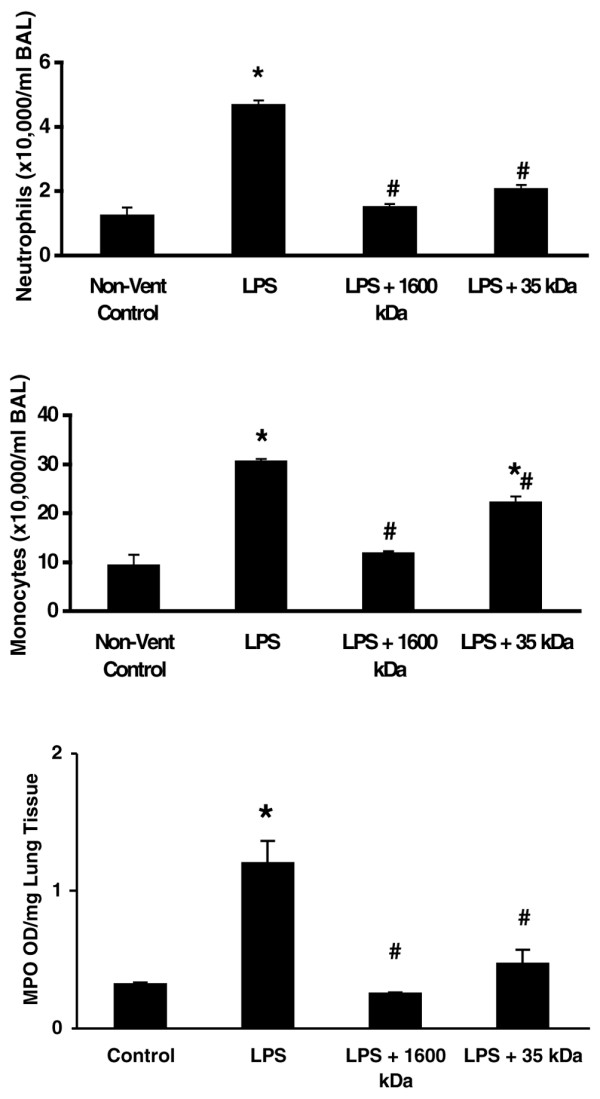
Only high-molecular-weight hyaluronan (1,600 kDa) completely blocked both lung neutrophil and monocyte infiltration. Animals were pretreated with either 1,600 kDa or 35 kDa hyaluronan 18 hours prior to ventilation. Lipopolysaccharide (LPS) (1 mg/kg) was given by arterial injection 1 hour prior to the start of ventilation. After 4 hours of ventilation, animals were sacrificed and the left lung was lavaged. **(a) **Neutrophils × 10,000/ml bronchoalveolar lavage fluid (BAL). **(b) **Monocytes × 10,000/ml BAL. **(c) **Myeloperoxidase assay optical density (MPO OD)/mg lung tissue. *P < 0.01 versus control, #*P *< 0.01 versus with LPS. Non-vent, nonventilated.

Rats receiving LPS had increased BAL neutrophils as compared with rats without LPS treatment. Pretreatment with HMW HA (1,600 kDa) decreased BAL neutrophils and the total lung neutrophil infiltrate with LPS. The results were similar with the use of 35 kDa HA (Figure 1). Lung neutrophil infiltration was confirmed with the myeloperoxidase assay (Figure 1). Only 1,600 kDa HA, and not 35 kDa HAcompletely blocked the increase in BAL monocytes. With LPS, 35 kDa HA only partially blocked BAL monocyte infiltration (Figure 1).

### Pretreatment with HMW HA (1,600 kDa) reduced the pathologic evidence of lung injury induced by lipopolysaccharide lung injury

**Figure 2:**
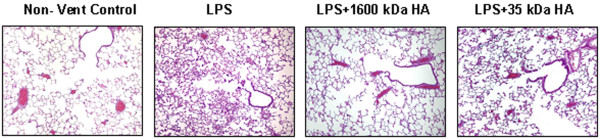
Only high-molecular-weight hyaluronan (1,600 kDa) decreased lung injury on pathology. Animals were pretreated with either 1,600 kDa or 35 kDa hyaluronan 18 hours prior to ventilation. Lipopolysaccharide (LPS) (1 mg/kg) was given by arterial injection 1 hour prior to the start of ventilation. After 4 hours of ventilation, animals were sacrificed and the left lung was fixed with formaldehyde, sliced and stained with hematoxylin and eosin. LPS caused edema and inflammatory cell infiltration, which was decreased with high-molecular-weight hyaluronan pretreatment. Non-vent, nonventilated.

**Figure 3:**
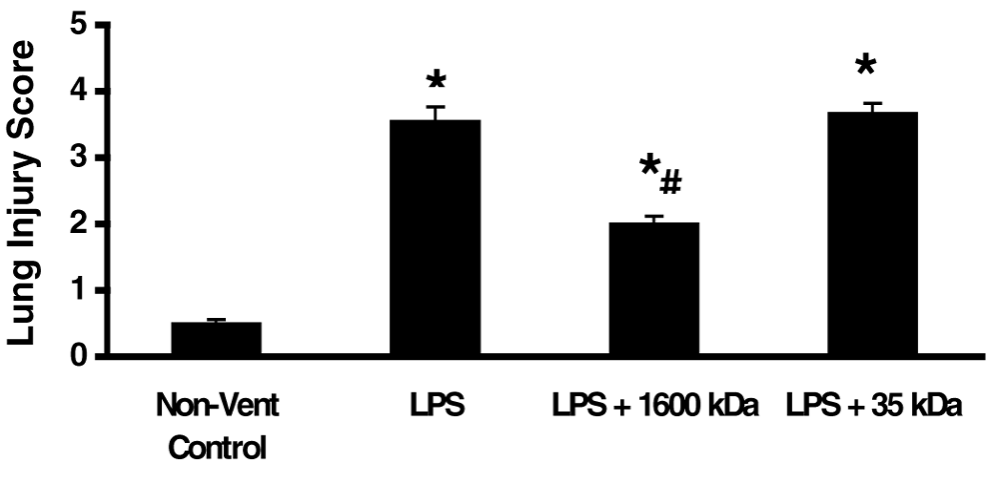
Only high-molecular-weight hyaluronan (1,600 kDa) significantly decreased the lung injury score. Animals were pretreated with either 1,600 kDa or 35 kDa hyaluronan 18 hours prior to ventilation. Lipopolysaccharide (LPS 1 mg/kg) was given by arterial injection 1 hour prior to the start of ventilation. After 4 hours of ventilation, animals were sacrificed and the left lung was fixed with formaldehyde, sliced and stained with hematoxylin and eosin. Lung injury, measured by the lung injury score as described in Materials and methods, showed substantial protection by 1,600 kDa hyaluronan but not 35 kDa hyaluronan. *P < 0.01 versus control, #*P *< 0.01 versus LPS. Non-vent, nonventilated.

Rats with LPS had poor alveolar distention and collapse, had intense inflammatory cell infiltration in the interstitium, had thickened perivascular clubbing and had alveolar hemorrhage on pathology slides (Figure 2). The lung injury score of the rats receiving LPS was significantly greater compared with rats without LPS (Figure 3). We found that rats with LPS receiving 1,600 kDa HA pretreatment, but not 35 kDa HA pretreatment, had less evidence of lung injury on pathology and had significantly decreased lung injury scores (Figures 2 and 3). The inhibition of lung injury by 1,600 kDa HA and not 35 kDa HA correlated with 1,600 kDa HA inhibition of IL-1β.

### Pretreatment with both 1,600 kDa and 35 kDa hyaluronan inhibited lipopolysaccharide-induced MIP-2 and TNFα mRNA and protein production with mechanical ventilation

**Figure 4:**
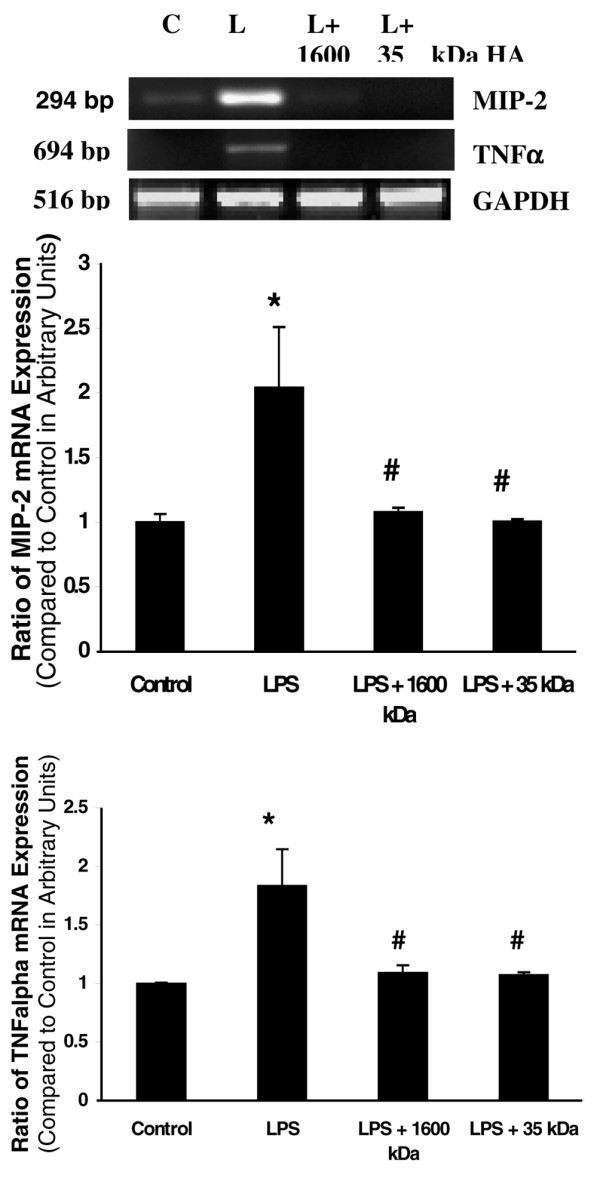
Hyaluronan (1,600 kDa and 35 kDa) inhibited cytokine mRNA expression. Animals were pretreated with either 1,600 kDa or 35 kDa hyaluronan 18 hours prior to ventilation. Lipopolysaccharide (LPS) (1 mg/kg) was given by arterial injection 1 hour prior to the start of ventilation. After 4 hours of ventilation, animals were sacrificed and the right upper lobe was flash frozen for extraction of RNA. Macrophage inflammatory protein-2 (MIP-2) and TNFα mRNA expression was decreased with high-molecular-weight hyaluronan or low-molecular-weight hyaluronan. **(a) **RT-PCR. **(b) **Quantitation of mRNA expression for MIP-2. **(c) **Quantitation of mRNA expression for TNFα. C, Control; L, LPS. *P < 0.05 versus control, #*P *< 0.05 versus LPS.

**Figure 5:**
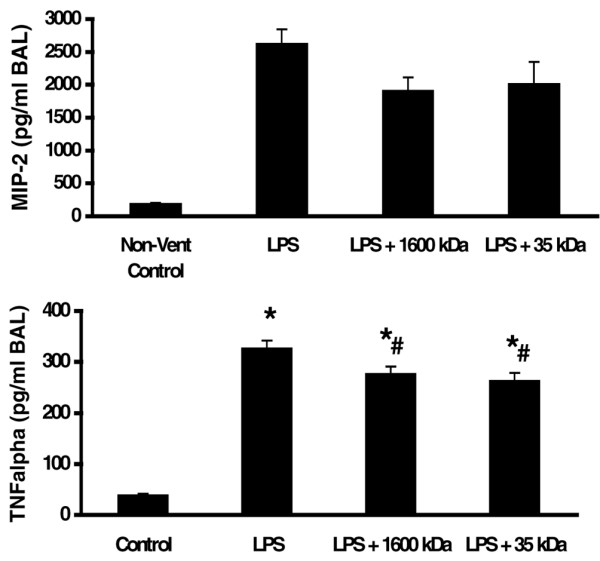
Pretreatment with hyaluronan (1,600 kDa and 35 kDa) inhibited cytokine protein expression. Animals were pretreated with either 1,600 kDa or 35 kDa hyaluronan 18 hours prior to ventilation. Lipopolysaccharide (LPS) (1 mg/kg) was given by arterial injection 1 hour prior to the start of ventilation. After 4 hours of ventilation, animals were sacrificed and the left lung was lavaged. Cytokine protein expression in bronchoalveolar lavage fluid (BAL) when measured by ELISA. Macrophage inflammatory protein-2 (MIP-2) and TNFα protein expression was decreased with high-molecular-weight hyaluronan or low-molecular-weight hyaluronan. **(a) **MIP-2. **(b) **TNFα. *P < 0.05 versus control, #*P *< 0.05 versus LPS. Non-vent, nonventilated.

In a comparisonn between rats with and without LPS at the same tidal volume, rats with LPS showed increased MIP-2 and TNFα mRNA and protein levels compared with rats without LPS. Rats receiving either 1,600 kDa HA or 35 kDa HA pretreatment showed less MIP-2 and TNFα production at the mRNA and protein level compared with rats with sepsis (Figures 4 and 5).

### Systolic blood pressure and heart rate in rats treated with HMW HA (1,600 kDa) 1 hour post lipopolysaccharide infusion

To further investigate the use of HMW HA infusion as a treatment for sepsis-induced lung injury, HMW HA (0.025%, 0.5% and 0.1%) was infused at 0.5 ml/hour starting at the time of ventilation. For these experiments, the maximum dose of LPS (3 mg/kg) that allowed survival of the animals was used. Saline was infused as needed to maintain a systolic pressure of about 70 mmHg to eliminate the confounding effects of hypotension. The systolic blood pressure was not significantly different between groups.

The heart rate was significantly higher in animals exposed to LPS, there were no significant differences between HMW HA-treated groups, and animals treated with CMC had a higher heart rate than those exposed to 0.1% HMW HA and than control animals (Table 2). All doses of HA and CMC increased the arterial oxygen pressure levels significantly (*P *< 0.05) above LPS-exposed animals (LPS, 51 ± 10 mmHg; LPS + 0.025% HA, 84 ± 7 mmHg; LPS + 0.05% HA, 67 ± 3 mmHg; LPS + 0.1% HA and LPS + 0.1% CMC, 75 ± 7 mmHg).

**Table 2:** Hemodynamics in the treatment model

	Systolic blood pressure		Heart rate	
	
Group	Baseline	4 hours	Baseline	4 hours
Control	125 ± 6	88 ± 6	425 ± 25	327 ± 9
LPS alone	98 ± 8	70 ± 5	465 ± 11	438 ± 28*
LPS + 0.025% of 1,600 kDa HMW HA	125 ± 5	72 ± 8	442 ± 21	448 ± 9*
LPS + 0.5% of 1,600 kDa HMW HA	112 ± 9	86 ± 6	475 ± 17	445 ± 10*
LPS + 0.1% of 1,600 kDa HMW HA	97 ± 16	87 ± 6	495 ± 18	401 ± 23*
LPS + 0.1% sodium carboxymethyl cellulose	97 ± 8	85 ± 7	464 ± 18	474 ± 30* ^†^

There were no statistical differences between groups for systolic blood pressure. In the lipopolysaccharide (LPS)-exposed groups the heart rate was statistically higher, but there were no statistical differences between the high-molecular-weight hyaluronan (HMW HA) treatment groups. LPS + 0.1% sodium carboxymethyl cellulose was statistically higher than LPS + 0.1% HMW HA. *P < 0.01 versus control. ^†^*P *< 0.01 versus LPS + 0.1% HMW HA.

### Treatment with HMW HA (1,600 kDa) 1 hour post lipopolysaccharide infusion blocked both lung neutrophil infiltration and acute lung injury in a dose-dependent manner

**Figure 6:**
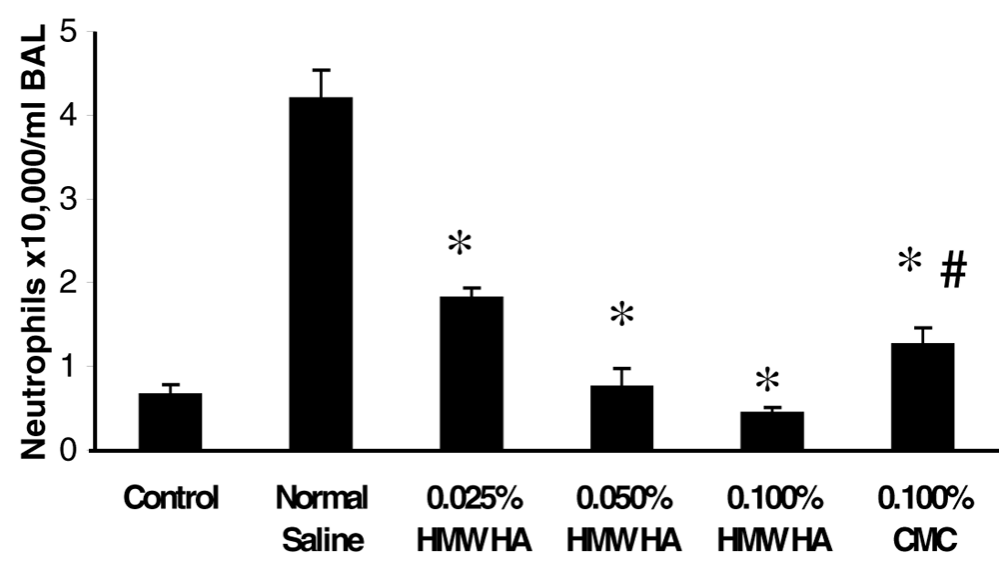
High-molecular-weight hyaluronan (1,600 kDa) given after lipopolysaccharide infusion blocked neutrophil infiltration in a dose-dependent manner. Animals were infused with 0.025%, 0.05%, or 0.1% high-molecular-weight hyaluronan (HMW HA) or 0.1% sodium carboxymethyl cellulose (CMC) at 500 μl/hour starting 1 hour after lipopolysaccharide (LPS) (3 mg/kg) infusion. Intravenous HMW HA given post LPS infusion showed a dose-dependent decrease in neutrophil infiltration, which was only partially explained by charge – as evidenced by significantly less inhibition with 0.1% CMC, a positively charged carbohydrate of similar molecular weight, as compared with 0.1% HMW HA. *P < 0.05 versus LPS, #*P *< 0.05 versus LPS + 0.1% HMW HA. BAL, bronchoalveolar lavage.

**Figure 7:**
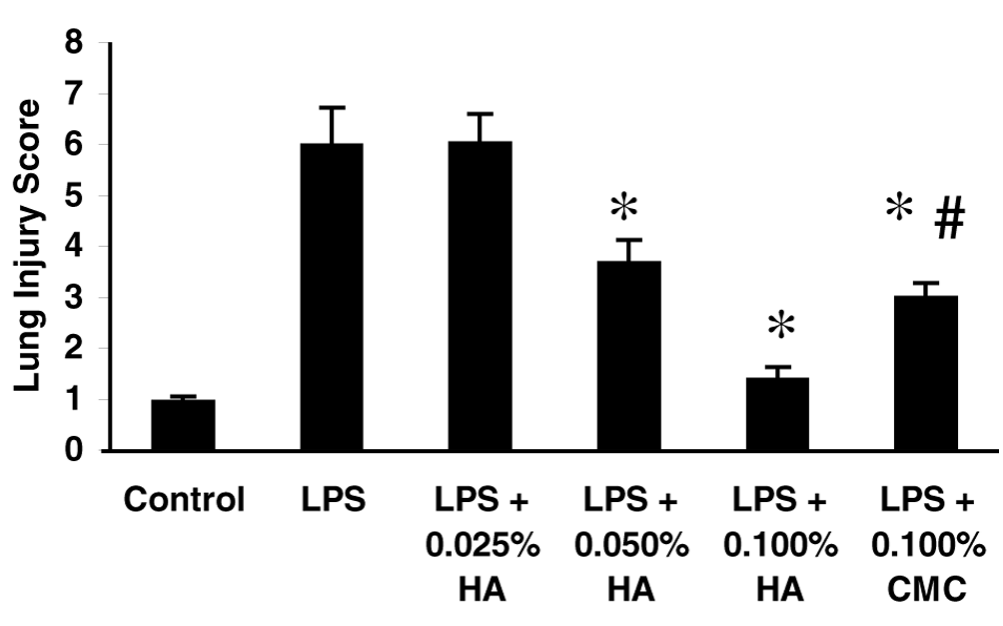
High-molecular-weight hyaluronan (1,600 kDa) given after lipopolysaccharide infusion blocked lung injury in a dose-dependent manner. Animals were infused with 0.025%, 0.05%, or 0.1% high-molecular-weight hyaluronan (HMW HA) or 0.1% sodium carboxymethyl cellulose (CMC) at 500 ml/hour starting 1 hour after lipopolysaccharide (LPS) infusion. *P < 0.05 versus LPS, #*P *< 0.05 versus LPS + 0.1% HMW HA.

HMW HA inhibited neutrophil infiltration (Figure 6), MIP-2 mRNA expression (data not shown), and acute lung injury scores (Figure 7) in a dose-dependent manner. To evaluate whether this effect was secondary to the positive charge of HA or to the anti-inflammatory properties of HA, 0.1% CMC was infused at 0.5 ml/hour. CMC at an equal concentration to HMW HA (0.1%) partially blocked lung neutrophil infiltration (Figure 6) and lung injury (Figure 7), but not to the same extent as HMW HA – suggesting that the positive charge of HMW HA was at least partially responsible for the therapeutic effects.

Post-treatment with intraperitoneal HMW HA failed to protect against inhalation acute lung injury, and therefore was not used in the present study (data not shown)

## Discussion

In the present study we investigated whether administration of exogenous HMW HA could be used as a therapy for sepsis-induced acute lung injury with mechanical ventilation. We demonstrated that pretreatment with HMW HA (1,600 kDa) inhibited inflammatory cell infiltration, cytokine production, and lung injury with mechanical ventilation. LMW HA (35 kDa) inhibited lung neutrophil infiltration and cytokine production, but did not inhibit lung injury or lung monocyte infiltration. HMW HA used in a therapeutic manner 1 hour after LPS infusion inhibited LPS-induced lung inflammation and lung injury in a dose-dependent manner.

HMW HA is an effective treatment in a variety of disease conditions. HMW HA has been shown to be a beneficial treatment for osteoarthritis. HMW HA can downregulate proinflammatory cytokines including IL-8, TNFα, and inducible nitric oxide synthase in fibroblast-like synoviocytes [18]. HMW HA prevented acute liver injury by reducing plasma MIP-2, TNFα, and IFNγ in a T-cell-mediated liver injury mouse model [11]. HMW HA has been shown to be protective in animal models of emphysema, can decrease the number of acute infections in chronic bronchitis in humans, can block group A streptococcus colonization in mice, can block pancreatic elastase-induced bronchoconstriction and neutrophil elastase-induced airway responses in sheep, can decrease peritoneal permeability secondary to infection in rats, and can reduce exercise-induced airway hyperreactivity in humans [19-23]. Beneficial effects in sepsis, however, have not been previously demonstrated.

Our findings are consistent with previous observations that mice overexpressing HMW HA are protected from bleomycin-induced lung injury [9]. Both HAS 1 and HAS 2 produced HMW HA. HASs are located on the cell surface and secrete the chains of HA into the extracellular matrix [1]. We have found in the normal lung that HA is of the HMW HA form; however, in high-tidal-volume-induced lung injury in rats we found both HMW HA and LMW HA.

To establish whether HMW HA could potentially have beneficial effects in the treatment of sepsis we initially used pretreatment with an intraperitoneal injection of 35% HMW HA prior to LPS injection. This concentration, given intraperitoneally 18 hours before liver injury, has been shown to be absorbed into the systemic circulation and to prevent concavalin-induced liver injury [11]. We then explored the use of HMW HA as a treatment for sepsis-induced lung injury. We had in previous studies found that HMW HA given intraperitoneally after smoke inhalation failed to protect lung injury (data not shown), probably related to delayed absorption, and 35% HMW HA was too viscous for intravenous injection. We therefore used continuous infusion of 0.025%, 0.05% and 0.1% HMW HA, concentrations that allowed intravenous infusion, starting 1 hour after injection of LPS.

We used intra-arterial LPS rather than the more conventional intravenous route of injection. We designed our model to produce lung injury over a 4-hour period that did not result in death but in a lung injury that was increased by high-tidal-volume ventilation over this period. We studied both venous and arterial injection. With arterial injection we found that there was increased neutrophil infiltration with arterial injection (61 × 10^3 ^± 10 cells/ml BAL fluid) than with venous injection (23 × 10^3 ^± 1 cells/ml BAL fluid, *P *< 0.05). Based on this response we chose the intra-arterial route. Intra-arterial injection has been used in other models of sepsis [24,25].

The difference in effects on acute lung injury scores between HMW HA and LMW HA may have been related to the different effects of the two molecular weights. HMW HA may block the effects of LMW HA produced in lung injury. The breakdown of HMW HA causes HA fragments to increase quickly and markedly in response to endotoxin [26], and elevated levels of plasma HA fragments have been detected in patients with septicemia [27]. LMW HA (200 kDa) isolated from the serum of patients with acute lung injury stimulated cytokine production in macrophages [9]. LMW HA mediates bleomycin-induced lung injury [9,28-31].

In previous studies, we demonstrated that *de novo *synthesis of LMW HA by HAS 3 was induced in lung fibroblasts exposed to cyclic stretch via tyrosine kinase signaling pathways [7]. *In vivo*, very-high-tidal-volume ventilation (30 ml/kg) induced LMW HA production, was dependent on HAS 3, and resulted in increased neutrophil infiltration in the lungs of mice [8]. Alternatively, the beneficial effects of HMW HA inhibition on inflammation may have been secondary to an increase in the ratio of HMW HA to LMW HA, thereby maintaining the level of HMW HA in the extracellular matrix and maintaining the integrity of the extracellular matrix [4,29].

HA receptors include CD44, RHAMM, Toll-like receptor 2 and Toll-like receptor 4 [9,32-34]. LMW HA induces cytokine production by binding to HA cell surface receptors. LMW HA (200 kDa) isolated from the serum of patients with acute lung injury stimulated cytokine production by binding to Toll-like receptor 2 and Toll-like receptor 4. LMW HA binding to Toll-like receptor 2 and Toll-like receptor 4 initiates mRNA expression by activation of the JNK pathways and through MyD88 activation [9,33,34]. HMW HA has been shown to block the action of LMW HA by competing LMW HA binding to its receptors [35]. The beneficial effects of HMW HA in this model of sepsis may have been secondary to HMW HA blocking the binding of LPS or LMW HA to Toll-like receptors, which mediate inflammation.

Surprisingly, infusion of LMW HA – at the size (35 kDa) and concentration (up to 1%) used in the present study – did not cause increased inflammation, and actually inhibited lung inflammation. LMW HA has been found to be proinflammatory by many authors, but not in all studies. Other authors have found that it is the protein and DNA contamination found in the LMW HA that is proinflammatory, and not the LMW HA itself [10,36]. One explanation of the lack of proinflammatory effects of the HA used in this study is the purity of the compound. The HA used in the present study has <0.1% protein and <0.1 absorbance units of neucleotides. We cannot rule out longer exposures or higher concentrations of LMW HA or other sizes of LMW HA causing inflammation. Since the 35 kDa LMW HA failed to prevent acute lung injury on pathology in our pretreatment studies, we did not use LMW HA in the postinjury studies.

Both HMW HA and LMW HA inhibited MIP-2 production in the BAL and inhibited infiltration of neutrophils and monocytes into the lung. The inhibition of MIP-2 most probably accounts for this effect. It has been previously shown that a gradient of chemokines between the alveoli and the circulation is needed to induce migration into the alveolar space [15]. We have previously shown that neutralization of MIP-2 in the airways prevents lung inflammatory cell infiltration in ventilator-induced lung inflammation [13].

Alternatively the beneficial effects were not secondary HMW HA inhibition of LMW HA, but secondary to an increase in the ratio of HMW HA to LMW HA – thereby maintaining the level of HMW HA in the extracellular matrix and maintaining the integrity of the extracellular matrix [4], and preventing the influx of inflammatory cytokines into the alveoli. This maintenance of the extracellular matrix may be an important mechanism in the prevention of acute lung injury by HMW HA.

Infusion of CMC, a carbohydrate with a positive charge similar size to the HMW HA, was used to control for the effects of charge. CMC blocked inflammation and lung injury – but not to the same extent as HMW HA. Since LMW HA and CMC partially blocked lung inflammation, the positive charge of HA may play a role in preventing lung injury by binding negatively charged inflammatory proteins.

One limitation of our study comparing LMW HA and HMW HA is the difference in molarity between the two infusions. We were unable to match molarity with the two infusions, since the high concentration of LMW HA that would be necessary to match the molarity between the solutions was not soluble. We cannot rule out that the beneficial effects of HMW HA may be due to the higher molarity of the solution being a better method of fluid resuscitation. We used additional saline infusions, however, to maintain the systolic blood pressure above 70 mmHg to eliminate hypotension as a confounding factor. The systolic blood pressure did not differ between groups.

An important part of our model is the use of mechanical ventilation with LPS infusion. The management of acute respiratory failure requires the use of positive-pressure mechanical ventilation to provide adequate ventilation and oxygenation. But mechanical ventilation with a high tidal volume leads to ventilator-induced lung injury by alveolar overdistention coupled with repeated collapse and reopening during mechanical ventilation, which initiates a cascade of proinflammatory cytokines. Even mechanical ventilation with moderate tidal volumes can augment the sepsis-induced lung injury by synergistically increasing lung cytokines, and may play a pivotal role in the development of acute lung injury in patients with sepsis [37-40]. The augmentation of acute lung injury by high tidal volumes has been termed ventilator-associated lung injury.

## Conclusion

HMW HA attenuated both lung inflammation and the extent of lung injury in a rat model of sepsis with mechanical ventilation, whereas LMW HA only inhibited lung inflammation and not the acute lung injury scores. These findings of the beneficial effects of HA in sepsis-induced lung injury are intriguing and warrant further investigation. Since LMW HA and CMC, a carbohydrate with a positive charge, also partially blocked lung inflammation, the size of HA may not be the only factor involved in the prevention of lung inflammation.

## Key messages

• HMW HA can inhibit acute lung injury secondary to sepsis.

• LMW HA was not as effective in inhibiting acute lung injury, but did inhibit inflammation.

• The mechanism of HMW HA inhibition of acute lung injury may be secondary to the positive charge of the molecule as well as to the size of the molecule.

## Abbreviations

BAL: bronchoalveolar lavage; CMC: sodium carboxymethyl cellulose; ELISA: enzyme-linked immunosorbent assay; HA: hyaluronan; HAS: hyaluronan synthase; HMW HA: high-molecular-weight hyaluronan; IFN: interferon; IL: interleukin; JNK: c-Jun NH_2_-terminal kinase; LMW HA: low-molecular-weight hyaluronan; LPS: lipopolysaccharide; MIP-2: macrophage inflammatory protein-2; PCR: polymerase chain reaction; RT: reverse transcriptase; TNF: tumor necrosis factor.

## Competing interests

HGG, CAH, and DAQ initiated a patent application for the use of HMW HA in the treatment of sepsis. DAQ received an unrestricted grant for the support of this work. DAQ now works for Novartis Pharmaceuticals, who did not support this work and are not involved in this work in any way. AS is employed by the Genzyme Corporation, who supported the patent application. All other authors declare that they have no competing interests.

## Authors' contributions

Y-YL and C-HL should be considered co-first authors: Y-YL is responsible for the work with pretreatment with hyaluronan, and C-HL is responsible for the work on hyaluronan as a therapeutic agent. Y-YL was responsible for carrying out the experiments and for data analysis in the pretreatment experiments. C-HL was responsible for carrying out the experiments and for data analysis in the therapeutic treatment experiments. HZ was responsible for the analysis for the PCR measurements. RD carried out the PCR measurements and performed the analysis under the guidance of HZ. AS provided all of the HA used in the experiments. OS oversaw the animal experiments, instructed Y-YH and C-HL in their implementation, and supervised the procurement and processing of the histology and myeloperoxidase assays. HGG is an expert in hyaluronan experiments and assisted in the experimental design. CAH is an expert in pulmonary physiology, assisted in the experimental design, and assisted in the data analysis and interpretation. DAQ is the principal investigator who initiated the project, designed the experiments, and oversaw the interpretation of the data. HM was responsible for performing and analyzing the control experiments. P-HM assited in the the assessment of the pathology.
